# Supported Web-Based Guided Self-Help for Insomnia for Young People Attending Child and Adolescent Mental Health Services: Protocol for a Feasibility Assessment

**DOI:** 10.2196/11324

**Published:** 2018-12-13

**Authors:** Bethany Cliffe, Abigail Croker, Megan Denne, Paul Stallard

**Affiliations:** 1 Department for Health University of Bath Bath United Kingdom; 2 Child and Family Service Oxford Health National Health Services Foundation Trust Keynsham United Kingdom

**Keywords:** adolescents, mental health, sleep, cognitive behavioral therapy, mobile phone

## Abstract

**Background:**

Sleep disturbance in adolescents is common, with up to one-third reporting significant symptoms of insomnia. Research with adults has demonstrated that Web-based cognitive behavioral therapy for insomnia (CBTi) can improve both sleep and mental health. However, research with adolescents is lacking, and we know little about whether CBTi would have similar effects on this younger population.

**Objective:**

This paper summarizes the protocol of a study to assess the feasibility of adding supported Web-based CBTi to the usual care of young people aged 14-17 years attending specialist Child and Adolescent Mental Health Services (CAMHS).

**Methods:**

This is an open trial where we will recruit young people (N=50) aged 14-17 years attending specialist CAMHS with primary or comorbid symptoms of insomnia. In addition to their usual care, young people will be provided with Sleepio, a 6-session, Web-based CBTi self-help program for insomnia. Sleepio teaches a range of techniques including sleep hygiene, relaxation training, stimulus control, sleep restriction, and cognitive techniques that participants will be helped to apply through brief, weekly telephone support calls. Questionnaires and interviews will be completed at baseline and postintervention (8-10 weeks) and will assess sleep, symptoms of depression and anxiety, and acceptability of Sleepio and telephone support.

**Results:**

Recruitment started in May 2018 and continued until the end of October 2018.

**Conclusions:**

This study will provide preliminary evidence about whether supported Web-based CBTi is acceptable to young people with mental health problems and about the postintervention effects on sleep and symptoms of anxiety and depression. This information will determine whether a randomized trial to determine the effectiveness of Sleepio should be undertaken.

**International Registered Report Identifier (IRRID):**

DERR1-10.2196/11324

## Introduction

### Insomnia

Poor sleep during adolescence is common, with insomnia, defined as chronic dissatisfaction with sleep quantity or quality, being the most prevalent sleep disorder [1,2]. Insomnia symptoms are reported by one-third of adolescents and up to a quarter fulfill the diagnostic criteria for insomnia, depending on the definition and method of assessment [3,4]. Insomnia symptoms are persistent [2] and are associated with significant mental health problems including depression, anxiety, substance abuse, and suicidal ideation [3,5-7].

### Association Between Insomnia and Mental Health

Research examining the nature of association between adolescent sleep disturbance and mental health is limited and the findings are inconsistent [8]. There is evidence of a bidirectional relationship, where symptoms of insomnia during adolescence both predict and are predicted by depression and depressive symptoms [9-11]. However, overall, there is more evidence to suggest that insomnia symptoms precede the development of anxiety and depression in adolescence more than the reverse [8,12-14]. This suggests that the provision of interventions that directly address insomnia could reduce the risk of developing mental health problems or reduce current symptomatology [12].

### Cognitive Behavioral Therapy for Insomnia

With adults, there is well-established evidence that treating insomnia can improve mental health including depression [15], anxiety [16], and psychotic experiences [17]. Interventions are based on cognitive behavioral therapy for insomnia (CBTi) and typically include a range of techniques including stimulus control, relaxation training, sleep restriction, sleep hygiene, and cognitive techniques to manage worries and intrusive thoughts [18]. Insomnia interventions can be delivered via the internet, with systematic reviews concluding that Web-based CBTi is effective and improves both sleep and mental health [19-21].

### Cognitive Behavioral Therapy for Insomnia for Adolescents

With adolescents, research examining the effect of CBTi on sleep and mental health is promising but very limited [18,22]. An open, uncontrolled pilot study assessing a 5-week CBTi intervention for depressed adolescents with insomnia found postintervention improvements in sleep and mood [23]. Similarly, augmenting depression treatment with CBTi in a randomized controlled trial involving 40 adolescents aged 12-20 years resulted in positive effects on sleep and depression [24]. In a community study, Bruin et al [25] found that CBTi delivered either face-to-face or over the internet to 12-19-year olds with insomnia was similarly effective and resulted in comparable improvements in sleep and psychopathology (anxiety and depression) compared with a waiting list control group. The authors concluded that improvements in psychopathology were attributable to a reduction of insomnia and recommended that further research should be undertaken within clinical settings.

### Study Aim

The aim of this study is to assess the feasibility of adding supported Web-based CBTi to the usual care for young people aged 14-17 years attending specialist Child and Adolescent Mental Health Services (CAMHS).

## Methods

### Study Design

This is a pre-post uncontrolled mixed-methods feasibility study. The study was funded by the Wiltshire Child Mental Health Commissioning Group, and ethical approval was obtained from the South West-Central Bristol Research Ethics Committee (17/SW/0178).

### Setting

The study will be undertaken in CAMHS within the Oxford Health National Health Services Foundation Trust. The Trust serves a wide geographical area that includes Bath and North East Somerset, Swindon, and Wiltshire.

### Participants

Young people will be eligible to participate if they are aged 14-17 years, attending CAMHS with symptoms of insomnia (ie, time asleep/time in bed ≤85%) as either a primary issue or as a comorbidity, motivated to try and improve their sleep, and interested in using Sleepio.

Motivation is assessed by rating each of 3 questions on a 10-point Likert scale from 0 (strongly disagree) to 10 (strongly agree). The questions relate to problem severity (“At present, sleep is a big problem for me”), desire to change (“I want to change my sleep”), and self-efficacy (“I feel I can change my sleep”). For inclusion, each item must be rated ≥5.

Young people will be ineligible to participate if they are presenting with active suicidal ideation, they have been diagnosed with psychosis, there are current safeguarding concerns (ie, the young person has suffered abuse within the last 6 months or is the subject of a safeguarding investigation), or they have a significant developmental disorder (eg, autism) that prevents them from understanding the program.

As this is a feasibility study, any face-to-face intervention or medication that the young person is receiving through CAMHS will not be interrupted. This trial will, therefore, run alongside any treatment as usual.

### Recruitment and Consent

Clinical staff working in CAMHS teams across Bath and North East Somerset, Swindon, and Wiltshire will identify eligible young people. Interested young people and their caregivers will be provided with a project information sheet, and their details will be passed to the research team. A research assistant will contact the young person to discuss the project, obtain written consent, and complete baseline measures. For those under the age of 16, parental consent will also be required. Meetings will take place at the individual’s home, at the University of Bath, via telephone, or at a community venue, depending on participants’ preference.

### Intervention

**Figure 1:**
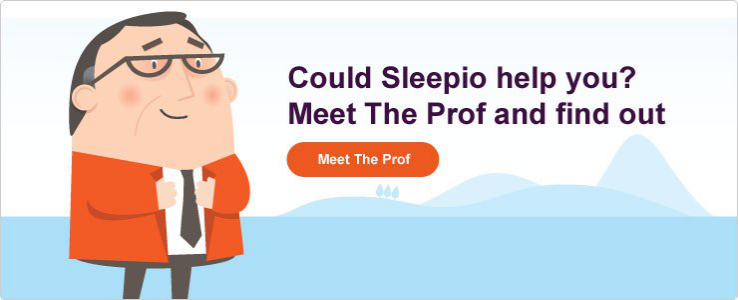
Sleepio's animated therapist, The Prof. Source: Big Health licensed under fair use.

**Figure 2:**
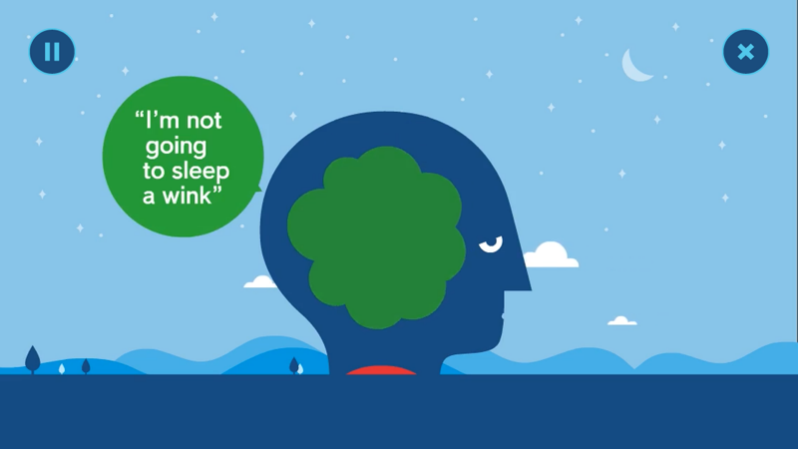
Part of a Sleepio session around debunking myths about sleep. Source: Big Health licensed under fair use.

Sleepio is an established, fully automated, Web-based, self-administered sleep intervention that has been evaluated with adults [17,26,27]. The intervention is free to study participants and consists of 6 sessions, each lasting approximately 20 minutes, which are released each week. The sessions are accessed via a Web browser on a tablet, desktop, or mobile phone. The program is highly interactional, and content is presented via an animated cartoon therapist (The Prof). Participants complete daily mobile app or Web-based sleep diaries throughout the program, with an algorithm personalizing the content to the individual’s needs. Sleepio is based on CBTi and incorporates cognitive, behavioral, and educational components (Figures 1 and 2).

#### Cognitive Component

##### Paradoxical Intention

Insomnia sufferers often pay too much attention to their sleep and the consequences of sleep loss. Their worries about not being able to fall asleep increase their anxiety and arousal, which has the effect of inhibiting the onset of sleep. Paradoxical intention attempts to disrupt this unhelpful process. The person is encouraged to deliberately stay awake for as long as he or she can, thereby reducing the anxiety associated with attempting to sleep and resulting in an easier onset of sleep.

##### Cognitive Restructuring

People with insomnia tend to have unrealistic expectations and beliefs about sleep and the consequences of not sleeping. They may overestimate how much sleep they need, underestimate how long they have slept, or catastrophize about the consequences of poor sleep. Cognitive restructuring involves challenging these negative and unhelpful sleep beliefs and developing more balanced and helpful ways of thinking.

##### Mindfulness

Before falling asleep, insomnia sufferers report being very aware of their negative and unhelpful thoughts. They may ruminate about the previous night where they failed to sleep or rehearse worries about the future and how bad the next day will be if they do not sleep. Mindfulness techniques can help individuals to focus on the here and now and to acknowledge and accept their thoughts and feelings without engaging with them.

##### Positive Imagery

Sleep creates anxiety for insomnia sufferers, which contributes to their difficulty falling asleep. To counter this negative perception of sleep and the bedroom, individuals are encouraged to create positive images, thereby reducing anticipatory anxiety.

##### Putting the Day to Rest

Insomnia sufferers often complain of a racing mind, where they ruminate about their day and rehearse what they might need to do in the future. To counter this tendency, people are encouraged to make time before bed to reflect on the day and to plan for tomorrow so that they have emptied their minds before bed.

#### Behavioral Component

##### Sleep Restriction

If sleep is limited, there is a tendency to spend more time in bed in order to catch up. However, people with insomnia find it difficult to fall asleep, resulting in them spending more time in bed awake. Sleep restriction limits the amount of time in bed to maximize the proportion of time spent asleep. As sleep improves, the sleep restriction limit is increased.

##### Stimulus Control

This aims to strengthen the association between bed and sleep. Many insomnia sufferers spend a lot of nonsleep time in bed, tossing and turning, reading, watching TV, gaming, etc. Stimulus control involves establishing bed as a place for sleep. Using bed for nonsleep activities is discouraged, and if the person wakes or is unable to sleep after 15 minutes, he or she is encouraged to get up and do something else relaxing before trying again.

##### Relaxation

To help individuals feel more relaxed and ready for sleep, they are taught a range of relaxation exercises they can undertake. These are designed to relax their body by reducing the physiological sensations of stress, thereby preparing individuals for sleep.

#### Educational Component

##### Sleep Hygiene

This provides information about developing a calming nighttime routine and place to sleep. Information is provided about reducing caffeine, blue screen use, and daytime naps; increasing exercise; and making the bedroom warm, dark, quiet, and free from distractions.

##### The Process of Sleep

This helps discover some important information about the duration of normal sleep, the factors that affect both sleep quality and quantity, and the fact that sleep is an involuntary process.

There are four different areas of Sleepio: sleep diary, case file, library, and community. There is also a Sleepio app that can be used to augment the Web version. The app allows users to fill in their sleep diary, view their daily schedule, and access relaxation audio files.

#### Sleep Diary

Users are required to complete a daily Web-based sleep diary that feeds into the underlying algorithms that tailor the Sleepio program content to the individual.

#### Case File

At the very beginning of the program, users are asked to define the goals that they want to achieve with Sleepio. This progress is tracked throughout and can be viewed in individuals’ case files. Here, they can also view their to-do list and daily schedule that The Prof helps them compile throughout the course. The case file also contains tools such as a recommended reading list, a thought checker, and a day planner, and users can download worksheets and audio files.

#### Library

The library contains articles about sleep, which augment the Sleepio sessions.

#### Community

There is a community section within Sleepio where users can post comments and interact with each other. As this section is not moderated and is intended for adults, young people will be instructed not to access this area of the website.

#### Brief Telephone Support

Engagement with Web-based self-help mental health programs is variable and is typically lower than that with guided interventions [18]. Although Sleepio is a self-administered program, maintaining engagement and program compliance with CBTi may be particularly challenging for adolescents [19]. As Sleepio has not previously been used with this age group, we will augment the programs with brief (15 minutes), weekly support telephone calls from a trained Sleepio Assistant. The assistants have an undergraduate university degree in psychology and previous experience of working with children or young people. They have completed the Sleepio program themselves before undertaking a half-day training focusing on how to facilitate the program with young people. The support calls are designed to maintain motivation and engagement and follow a similar process to that used by Luik et al [27]. The calls will be empathic and motivating and will help the young person reflect on how techniques can be applied to their situation. The call will start by reviewing the young person’s sleep diary followed by a discussion about how any new strategies identified in previous Sleepio sessions have been applied. The discussion will then review the current Sleepio session and how and which new skills can be applied to his or her situation. Finally, the young person’s progress toward achieving his or her goals will be discussed, any questions answered, and the time and date of the next call agreed. The telephone calls are specifically restricted to sleep and the Sleepio program, and no other problems will be discussed. The length of each support call will be recorded.

### Study Procedures

**Figure 3:**
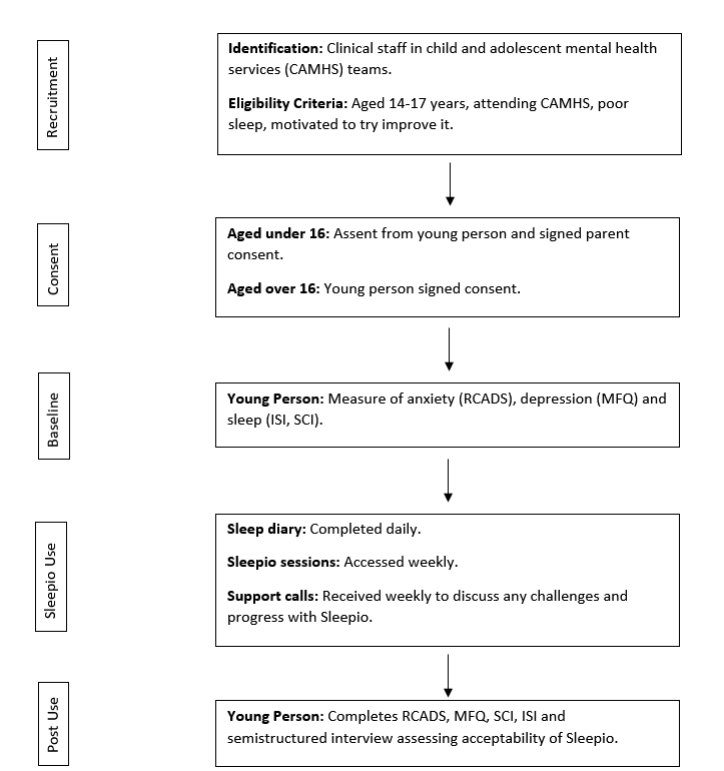
Study procedure. CAMHS: Child and Adolescent Mental Health Services; RCADS: Revised Child Anxiety and Depression Scale; MFQ: Mood and Feelings Questionnaire; ISI: Insomnia Severity Index; SCI: Sleep Condition Indicator.

The study procedures are summarized in Figure 3.

Once eligible young people have provided consent and completed baseline measures, they will be emailed an individual access code for Sleepio. A Sleepio Assistant will contact each young person to discuss the goals that they wish to achieve at the end of Sleepio and agree times for future support calls. Sessions are released each week.

The Sleepio Assistants will monitor progress through the Sleepio dashboard, which summarizes when the young person has accessed each session. Reminder emails and telephone calls will be sent if young people are not engaging with their session. When the 6 sessions have been completed, a member of the research team will arrange to meet with the young person and conduct the postuse assessment.

### Outcome Measures

The following standardized assessments will be completed pre- (baseline) and post-Sleepio completion (8-10 weeks). Although the program consists of 6 weekly sessions, previous research indicates that the majority of participants complete the course within 10 weeks [28]. Our primary outcome is changes in sleep, with secondary outcomes assessing symptoms of depression and anxiety.

#### Sleep

##### Insomnia Severity Index

The Insomnia Severity Index (ISI) is a 7-item self-report measure assessing symptoms of insomnia over a 2-week period on a 5-point scale. The ISI assesses the severity of sleep onset, sleep maintenance, and early morning awakening problems; sleep dissatisfaction; interference of sleep difficulties with daytime functioning; whether sleep problems are noticed by others; and distress caused by sleep difficulties [29].

##### Sleep Condition Indicator

The Sleep Condition indicator (SCI) is an 8-item self-report measure assessing sleep and its impact on daytime functioning over the previous month on a 4-point scale. The SCI is an internally consistent (alpha=.86) measure with a clinical cut-off <17 correctly identifying 89% of those with probable Diagnostic and Statistical Manual of Mental Disorders 5th edition insomnia disorder [26,30].

#### Mental Health

##### Anxiety: Revised Child Anxiety and Depression Scale

The Revised Child Anxiety and Depression Scale (RCADS) [31] is a 47-item questionnaire assessing Diagnostic and Statistical Manual of Mental Disorders 4th edition criteria for social phobia, separation anxiety, obsessive-compulsive disorder, panic disorder, generalized anxiety disorder, and major depressive disorder. Each item is rated on a 4-point Likert scale of frequency ranging from never (0) to always (3), and items are then summed to produce subscale and total anxiety scores. There are age- and gender-related norms for identifying clinically significant scores (total score ≥64-80).

##### Depression: The Mood and Feelings Questionnaire

The Mood and Feelings Questionnaire (MFQ) [32] consists of 33 items each rated as either true (scores 2), sometimes true (scores 1), or not true (scores 0). The MFQ has high criterion validity and correlates well with other measures of depression. A total score of ≥27 is associated with major depression, 17-26 with mild depression, and ≤16 with no mood disorder [32-34].

#### Experience of Sleepio

At the postuse assessment, a semistructured interview will be undertaken with young people to gather detailed feedback on their experience of Sleepio. The postuse interview collects both quantitative and qualitative data. Young people rate on a 4-point scale for ease of use, helpfulness, preference over face-to-face meetings, whether sessions were understandable, and if they would recommend Sleepio to a friend. Changes in sleep, mental health, and overall satisfaction are assessed on a 1-10 scale. Qualitative questions assess how, where, and how often Sleepio was accessed; which techniques and sessions were most useful; experience of telephone support; and how the program can be improved.

#### Usage

The number of Sleepio sessions completed by each individual will be recorded from the Sleepio dashboard along with his or her weekly sleep efficiency and quality ratings.

### Sample Size

Formal power calculations were not deemed necessary due to this being an initial feasibility study. Recruiting 50 young people should provide sufficient information to determine whether Sleepio is perceived as acceptable and results in improved sleep and mental health in young people [35].

### Statistical Analysis

We will present descriptive statistics summarizing the cohort in terms of age, gender, sleep, and anxiety and depressive symptomology. Descriptive statistics will also be used to summarize engagement with Sleepio in terms of the number of sessions completed versus the number of those who dropped out. *t* test analyses of mean scores will be conducted on the total scores for the pre- and postmeasures of sleep (ISI and SCI) and on the total and subscale scores for the pre- and postmeasures of mood (RCADS and MFQ). This will allow exploration of any changes in sleep or psychological functioning following Sleepio use. Postuse semistructured interviews will be analyzed to determine the acceptability of Sleepio among young people. The interviews will be audiorecorded and transcribed. A predefined framework will be derived from the interview schedule and adapted following participant responses for analysis.

## Results

The study closed at the end of October 2018.

## Discussion

### Study Aims

This study aims to determine the feasibility of adding supported Web-based CBTi for young people aged 14-17 years attending specialist CAMHS. Our study is addressing an important problem and will provide preliminary evidence about whether supported Web-based CBTi is acceptable to young people with mental health problems and about the postintervention effects on sleep and symptoms of anxiety and depression. If found to have a positive effect on mental health, this low-intensity intervention delivered with minimal therapist support could readily increase the limited capacity of traditional CAMHS. However, the implementation, effectiveness, and sustainability of supported CBTi in child mental health services would need to be determined before its widespread adoption could be advocated.

### Limitations

Although this is a feasibility study, limitations in the study design need to be acknowledged. First, we are relying on self-report measures of sleep and are not using objective actigraphy measures. Retrospective self-report may be prone to inaccuracies, but while wearable devices are able to prospectively monitor and track sleep, they are not always reliable or accurate [36]. Subjective reports do provide useful information, which we will supplement with prospectively completed sleep diaries as young people progress through Sleepio [8].

Second, this is an open trial, and we do not have any control or comparison groups. This will not allow comparative insights into whether any improvements are due to the Sleepio intervention, the young person’s ongoing mental health intervention, or the passing of time. At this stage, our primary aim is to determine the acceptability and feasibility of using Sleepio with young adolescents, not to determine its efficacy. The inclusion of comparison group(s) to control for these variables will be considered in a subsequent trial.

## Abbreviations

CAMHS: Child and Adolescent Mental Health Services
CBTi: cognitive behavioral therapy for insomnia
ISI: Insomnia Severity Index
MFQ: Mood and Feelings Questionnaire
RCADS: Revised Child Anxiety and Depression Scale
SCI: Sleep Condition Indicator
